# Olfactory Bulb Field Potentials and Respiration in Sleep-Wake States of Mice

**DOI:** 10.1155/2016/4570831

**Published:** 2016-05-10

**Authors:** Jakob Jessberger, Weiwei Zhong, Jurij Brankačk, Andreas Draguhn

**Affiliations:** Institute for Physiology and Pathophysiology, Heidelberg University, 69120 Heidelberg, Germany

## Abstract

It is well established that local field potentials (LFP) in the rodent olfactory bulb (OB) follow respiration. This respiration-related rhythm (RR) in OB depends on nasal air flow, indicating that it is conveyed by sensory inputs from the nasal epithelium. Recently RR was found outside the olfactory system, suggesting that it plays a role in organizing distributed network activity. It is therefore important to measure RR and to delineate it from endogenous electrical rhythms like theta which cover similar frequency bands in small rodents. In order to validate such measurements in freely behaving mice, we compared rhythmic LFP in the OB with two respiration-related biophysical parameters: whole-body plethysmography (PG) and nasal temperature (thermocouple; TC). During waking, all three signals reflected respiration with similar reliability. Peak power of RR in OB decreased with increasing respiration rate whereas power of PG increased. During NREM sleep, respiration-related TC signals disappeared and large amplitude slow waves frequently concealed RR in OB. In this situation, PG provided a reliable signal while breathing-related rhythms in TC and OB returned only during microarousals. In summary, local field potentials in the olfactory bulb do reliably reflect respiratory rhythm during wakefulness and REM sleep but not during NREM sleep.

## 1. Introduction

Neuronal network oscillations and their interactions are thought to be important for perception [1], sensory-motor coordination [2], and memory processes [3]. According to a recent hypothesis, the breathing rhythm serves as a clock for binding several orofacial senses to a common percept [4]. In line with this hypothesis, it was found that whisking synchronizes with breathing [5, 6] and that slow oscillations in the whisker barrel cortex of waking mice are locked to respiration [7]. Recently, we demonstrated in the dentate gyrus of anaesthetized [8] and awake [9] mice a respiration-related rhythm (RR) clearly distinct from theta oscillations. Discrimination of the RR from other slow rhythms requires registration of respiration, preferably as an electrical field potential which can be easily recorded together with other electrophysiological data. There are several other more direct ways to measure respiration in rodents. Intranasal pressure sensors or thermocouples implanted into the nasal cavity [10–12] are commonly used in rodents but are invasive devices which may compromise natural behavior. As a less invasive alternative, whole-body plethysmography [13–15] provides a reliable, state-independent biomechanical measure of respiration. However, plethysmography requires housing of the animals in specialized and closed small chambers, making it unpractical for use in combination with tethered brain recordings or complex behavioral tasks. For decades, it had been known that local field potentials (LFP) of the olfactory bulb (OB) of mammals are entrained by nasal respiration [16–19]. This signal has already been used to measure respiration rate in freely moving rats [20]. However, the reliability of OB LFP for estimating breathing and sniffing frequencies in mice has not been systematically studied and verified by alternative approaches, especially not through different states of vigilance. In the present study, we simultaneously used plethysmography, nasal thermocouple, and LFP recordings in OB during various sleep-wake states of mice.

## 2. Materials and Methods

### 2.1. Ethics Statement

All experiments were performed according to the guidelines of the European Science Foundation [21] and the US National Institutes of Health Guide for the Care and Use of Laboratory Animals (Institute of Laboratory Animal Research [22]) and were approved by the Governmental Supervisory Panel on Animal Experiments of Baden Württemberg at Karlsruhe (G84/13). All efforts were made to minimize animal suffering and to reduce the number of animals used.

### 2.2. Animal Care and Housing Conditions

C57BL/6N mice were purchased at 70 or 84 d of age from Charles River (Sulzfeld, Germany). Animals were housed in groups of four inside a ventilated Scantainer (Scanbur, DK) on an inverted 12 h/12 h light/dark cycle (light on 8:00 PM) for a minimum of 2 weeks. Mice had free access to food and water. After chronic electrode implantation, mice were kept individually throughout the experiment. The animals were killed with an overdose of isoflurane during brain perfusion.

### 2.3. Surgery

A total of 17 C57BL/6N mice (16 female and one male; 13–29 weeks old; weight: 22–27 g) were anesthetized with isoflurane in medical oxygen (4% isoflurane for induction, 1.5%–2.5% for maintenance, flow rate: 1 L per minute). For analgesia, 0.1 mg/kg of buprenorphine was injected subcutaneously before and 8 h after surgery (for details see [9]). After exposure of the skull, holes of 0.5–1.0 mm in diameter were drilled above the OB, the parietal cortex, and cerebellum according to stereotaxic coordinates; see below for coordinates based on [23]. Two stainless steel watch screws (1 × 3 mm) over the cerebellum served as ground and reference electrode. For monitoring the temperature of nasal air flow, precision fine bare wire thermocouples (80 *μ*m diameter, Omega Engineering, part #5TC-TT-KI-40-1 M) were implanted into both nasal cavities (−11 mm anteroposterior (AP), 0.5 mm mediolateral (ML)). A pair of varnish-insulated tungsten wires (50 *μ*m) was implanted into the granule cell layer of left OB (AP: +4.5; ML: 0.8; 1.3 mm ventral). For restraining the head of the animal, a steel pin (2.1 mm diameter, 14 mm length) was attached to the skull under an angle of 75 degrees.

### 2.4. Electrophysiology

One week after surgery, experiments began with a 1 h recording session in the plethysmograph (see below) or in a custom-built head-fixed setup with a treadmill for voluntary running [9]. Extracellular signals were filtered (1–500 Hz), amplified (RHA2000, Intan Technologies, Los Angeles, USA), digitized (2.5 kHz), and stored for offline analysis. A three-dimensional accelerometer mounted on the amplifier board at the animals head allowed movement detection. Mice habituated quickly to the head-restraining conditions and tolerated it well for up to 1 h. The whole-body plethysmograph (PG) consisted of a transparent cylindrical box (78 mm inner diameter, 165 mm height) which was connected to a reference chamber and adapted for collection of LFP (EMKA Technologies, SAS, France). Mice habituated well to the PG apparatus within the first session.

### 2.5. Data Analysis

**Figure 1:**
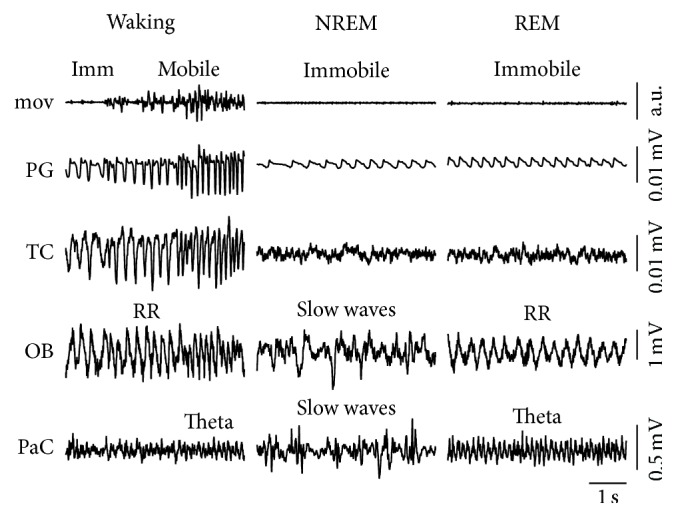
Examples of three vigilance states: waking, nonrapid eye moving (NREM), and rapid eye moving (REM) sleep. Three signals are sufficient for vigilance staging: (1) movement (mov) detection based on accelerometer activity; (2) detection of slow waves in parietal cortex (PaC), and (3) detection of theta (4–12 Hz) oscillations in the PaC reflecting activity in the underlying hippocampus. Imm: immobile; OB: olfactory bulb; PG: plethysmography; TC: thermocouple; RR: respiration-related rhythm.

Classification of vigilance states was based on (1) the level of accelerometer activity (Figure 1: mov; waking (Wk) > NREM, REM); (2) the amount of high amplitude slow wave activity in the neocortex (Figure 1: parietal cortex (PaC), slow waves; NREM > Wk, REM); (3) the amount of regular theta oscillations in the parietal cortex overlaying the dorsal hippocampus (Figure 1: PaC, theta; REM > NREM, active Wk > NREM). For detailed description of behavioral staging, see Brankačk et al. [24].

Data was analyzed in MATLAB (The Mathworks Inc., Natick, MA) using built-in and custom-written routines (for more details see [9]).

### 2.6. Spectral and Coherence Analysis

Power spectral density was calculated by means of the Welch periodogram method using the* pwelch.m* function from the Signal Processing Toolbox (50% overlapping, 4 s Hamming windows). Time-frequency power decomposition was obtained by means of the* spectrogram.m* function; sliding windows of 2 s and 50 ms time steps were used; phase* coherence* was obtained by means of the* mscohere.m* function from the Signal Processing Toolbox, using 2 s windows with 50% overlap. For all types of data analysis, LFP signals of 25 s duration were used.

### 2.7. Histology

After the experiments, animals were deeply anesthetized with isoflurane and perfused transcardially with PBS and subsequently with 4% paraformaldehyde (PFA). Brains were carefully dissected and stored in PFA overnight, and coronal sections were cut (50 *μ*m), mounted, and stained with cresyl violet. The electrode position was then verified by light microscopy.

### 2.8. Statistics

Data are expressed as mean ± SEM. For group comparisons of normally distributed data (Kolmogorov-Smirnov test), we used *t*-test or repeated measures ANOVA followed by Tukey's multiple comparison test. For data with non-Gaussian distribution, we used the nonparametric Friedman test, Mann-Whitney test, or the Wilcoxon signed rank test.

## 3. Results

### 3.1. In Awake Mice Plethysmography, Nasal Thermocouple and Olfactory Bulb Local Field Potentials Reveal Similar Respiration Signals

**Figure 2:**
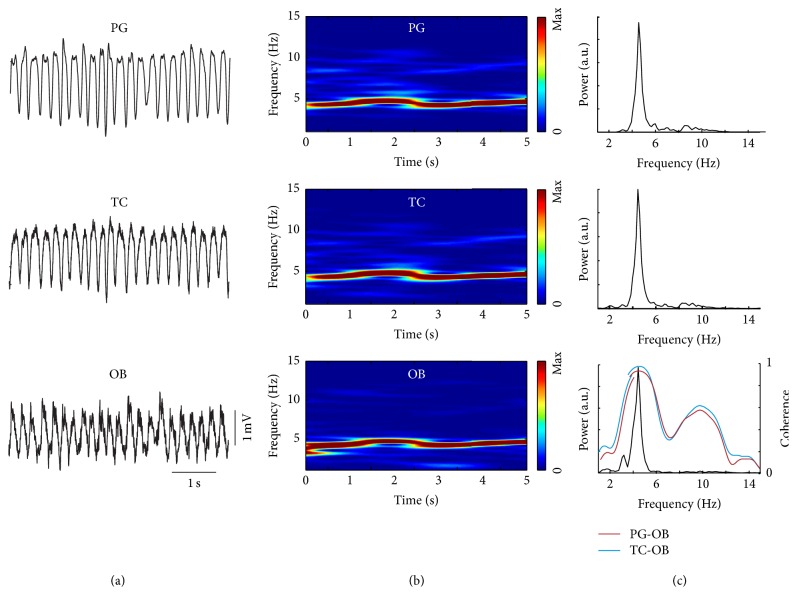
Plethysmography, nasal thermocouple, and olfactory bulb local field potentials similarly represent respiration during immobile waking. (a) Raw signals. (b) Time-frequency distribution. (c) Power spectral densities (black) and coherence (magenta: plethysmography (PG) versus olfactory bulb (OB), blue: thermocouple (TC) versus OB).

Respiration was recorded using a thermocouple (TC) chronically implanted into the nasal cavity. The TC signal reflects temperature changes of nasal air flow. Simultaneously, thoracic movements were detected with whole-body plethysmography (PG) indicating relative values of respiratory air flow (mL/s). In addition, local field potentials (LFP) from the granular cell layer of the olfactory bulb (OB) showed fluctuations entrained by nasal respiration as described earlier [18].  Figure 2 illustrates similarity of the three methods in a representative example of waking immobility (Figure 2(a): raw signals and Figure 2(b): time-frequency distribution). Note the respiration-related rhythm (RR) in the OB LFP. As expected, power spectral density analysis results in power peaks of identical frequency (4.42 Hz; Figure 2(c)). Phase coherence between PG and OB (Figure 2(c), lower graph, red) or TC and OB (Figure 2(c), lower graph, blue) was largest (~0.9) at the frequency of respiration with its harmonic at 8.84 Hz. Harmonics are also visible in power spectral densities of PG and TC.

### 3.2. During Waking the Power of Respiration-Related Rhythm in Olfactory Bulb Decreased with Increasing Respiration Rate

**Figure 3:**
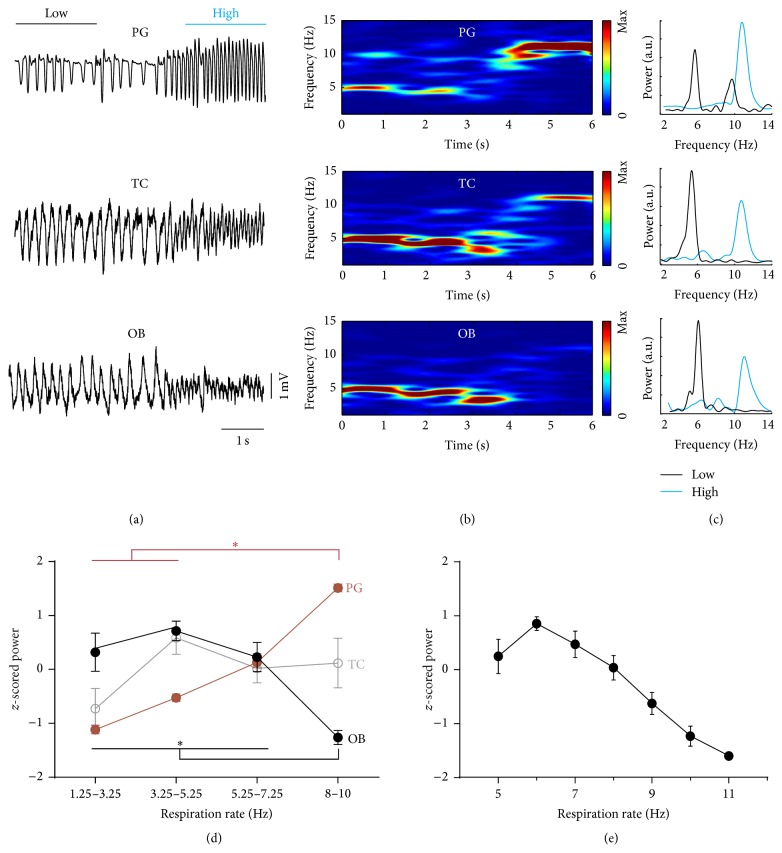
Breathing frequencies vary in freely moving animals. Transition from breathing to sniffing. (a) Raw signal. (b) Time-frequency distribution. (c) Power spectral density of the time periods indicated in (a). Power of olfactory bulb local field potential decreased with increasing respiration rate. (d) *z*-scored peak power of plethysmography (PG, red; *n* = 8; ^*∗*^ *p* < 0.0005; Friedman's test), thermocouple (TC, gray; *n* = 8; *p* = 0.22; repeated measures ANOVA), and LFP in olfactory bulb (OB, black; *n* = 8; ^*∗*^ *p* < 0.01; repeated measures ANOVA). (e) Negative correlation between OB peak power and respiration rate during running (*n* = 11; *r* = −0.958; *p* < 0.001, Pearson's correlation). Group results in (d) and (e) are shown as mean ± standard error of the mean (SEM).

We next investigated how peak power of the RR in OB varied with changing breathing frequency. Respiration rate in waking mice varies strongly, especially during interspersed spouts of sniffing [9, 11].  Figure 3 shows a representative example of an abrupt transition from slow breathing to fast sniffing. At high sniffing rates, the amplitude of the PG signal increased while the amplitude of the RR in OB decreased (Figure 3(a), raw signals). Despite the amplitude differences similar prominent rhythms were found for all three signals in the spectrograms (Figure 3(b)) and in power spectral density (PSD) plots, at low (Figure 3(c), black) and high frequencies (Figure 3(c), blue). For quantitative analysis, periods of waking were sorted into frequency bins of breathing/sniffing obtained by PG (Figure 3(d)). PSDs of the three simultaneously recorded respiration signals and coherence between PG and OB (not shown) were calculated for each interval. Peak powers of PG, TC, and RR in OB were *z*-scored and averaged. PG peak power increased with respiration frequency (Figure 3(d): red; *n* = 8; ^*∗*^ *p* < 0.0005; Friedman test), whereas TC power did not change (Figure 3(d): gray; *n* = 8, *p* = 0.22; repeated measures ANOVA). In contrast, peak power of the RR in OB decreased in the highest frequency bin (8 to 10 Hz) compared to the three remaining bins (Figure 2(d): black; *n* = 8; ^*∗*^ *p* < 0.01; repeated measures ANOVA; post hoc Tukey test). Mean coherence between PG and OB LFP did not change (not shown; *n* = 8; *p* = 0.15; repeated measures ANOVA). This indicates high coherence between OB, TC, and PG for all respiratory frequencies during waking.

### 3.3. Negative Correlation between Power of Respiration-Related Rhythm in OB and Breathing Frequency during Treadmill Running

To reduce respiratory variability, we next performed head-fixed experiments where the behavior of the mouse was basically clamped to either immobility or running on a circular treadmill and minimized sniffing [9]. Only periods of running were used here and divided into 1 Hz bins of increasing breathing frequency obtained by simultaneously recorded TC.  Figure 3(e) displays *z*-scored averages of RR peak power in OB (*n* = 11 mice) which linearly decreased with increasing respiration frequency (*n* = 7; *r* = −0.958; *p* < 0.001, Pearson's correlation).

### 3.4. Disappearance of the Respiration-Related Rhythm in Olfactory Bulb during NREM Sleep and Recurrence in REM Sleep

**Figure 4:**
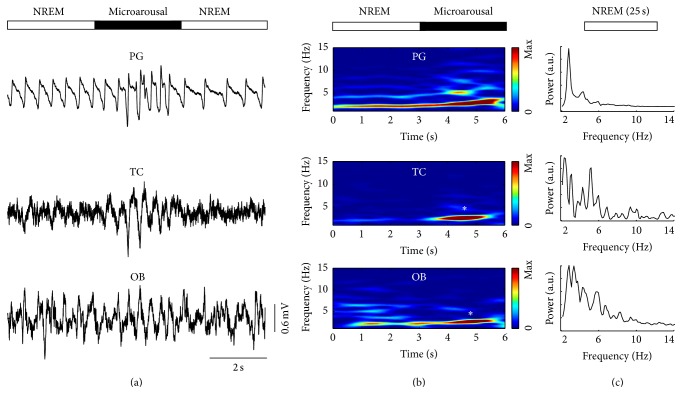
During NREM sleep only plethysmography reliably reflects respiration except during microarousals. (a) Raw signal of plethysmography (PG), thermocouple (TC), and olfactory bulb local field potential (OB). (b) Time-frequency distribution of the three signals shown in (a). (c) Power spectral density of NREM sleep reveals a clear peak in PG but no corresponding peaks in TC and OB. During microarousals (see asterisks), all three signals indicate respiration.

Large amplitude slow waves in the delta frequency range are characteristic features of nonrapid eye movement (NREM) sleep. Delta waves are superimposed on local field potentials in OB hiding traces of respiration-related fluctuations between 1.2 and 3.5 Hz which could be reliably detected with PG (see NREM in Figure 4(a), raw traces). Respiration-related signals in TC disappeared during NREM (Figures 4(a) and 4(b)), whereas the signal in PG remained. In contrast to the clear power peak in PG (Figure 4(c), upper panel), no distinctive peaks were discernible in spectrograms from TC and OB signals, respectively (Figure 4(c), middle and lower panel).

**Figure 5:**
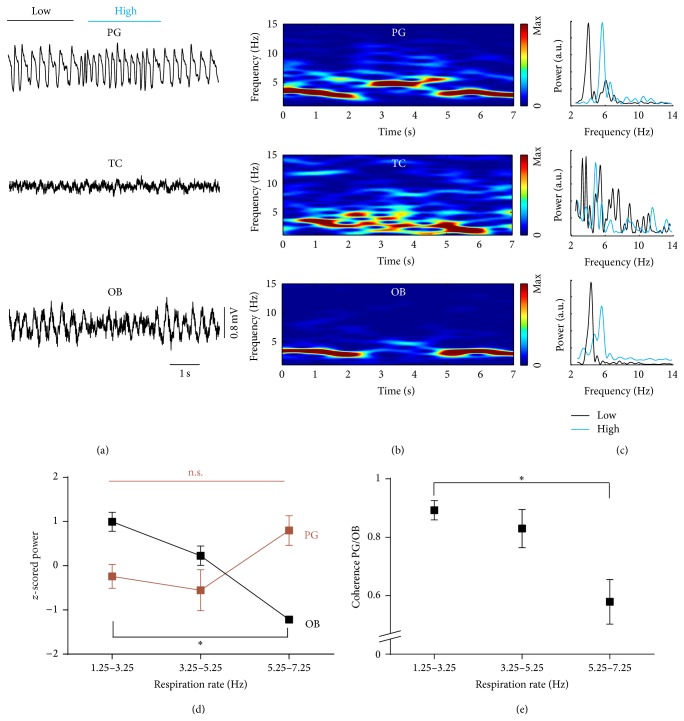
During REM sleep only plethysmography reliably indicates respiration for low and high breathing frequencies. Respiration-entrained local field potentials in olfactory bulb appeared mostly at low breathing frequencies. Thermocouple signal did not contain any respiration-related information. (a) Raw signals of plethysmography (PG), thermosensor (TS), and local field potential of the olfactory bulb (OB). (b) Time-frequency distribution. (c) Power spectral density of the time segments marked in (a). (d) *z*-scored averages of peak power (± SEM) of PG (red; *n* = 6; *p* = 0.1416; Friedman's test) and the LFP in OB (black; *n* = 6; ^*∗*^ *p* < 0.01; Friedman's test). (e) Coherence between PG and OB decreased with increasing breathing frequency (*n* = 6; ^*∗*^ *p* < 0.01; Friedman's test).

Both RR in OB and respiratory signal in TC regularly returned during microarousals (see asterisks in Figure 4(b)). During parts of REM sleep, especially at low breathing frequencies, RR in OB recovered (Figure 5(a), low; Figure 5(b)), whereas the TC signal remained absent. Clear power peaks were found for PG and OB for both low (Figure 5(c), upper and lower panel, black) and high (Figure 5(c), blue) breathing frequencies, in contrast to TC where no corresponding peaks were discernible in the power spectral density graph (Figure 5(c), middle panel). During REM sleep, RR in OB was detectable, but the signal was less reliable as compared to awake animals, especially at high breathing frequencies (Figures 5(a) and 5(c), lower panels, blue). For all corresponding bins of breathing frequency, RR peak power was smaller during REM compared to waking (1.25 to 3.25 Hz: *n* = 6; *p* < 0.05; *t*-test; 3.25 to 5.25 Hz: *n* = 6; *p* < 0.01; *t*-test; 5.25 to 7.25 Hz: *n* = 6; *p* < 0.05; Wilcoxon test). Figure 5(d) shows peak power averages of six animals of PG (red) and OB (black) recorded during REM sleep binned into three breathing frequency ranges as above. PG peak power did not depend on breathing frequency, in contrast to RR peak power in OB (Figure 5(d); black; *n* = 6; ^*∗*^ *p* < 0.01; Friedman's test). Likewise, coherence between PG and OB signals decreased between the lowest and highest breathing frequency bins (Figure 5(e); *n* = 6; ^*∗*^ *p* < 0.01; Friedman's test). Coherence between PG and OB was also lower in REM compared to waking (not shown) for the two highest frequency bins (*n* = 6; *p* < 0.05 and *p* < 0.01; Wilcoxon test). In summary, plethysmographic data provided the only reliable measure of respiration throughout NREM sleep and was more reliable during REM sleep at high breathing frequencies compared to OB LFP which was a good indicator of respiration during REM sleep with low respiration rates.

### 3.5. All Three Measurements of Respiration Are Coherent during Waking but Not in Sleep

**Figure 6:**
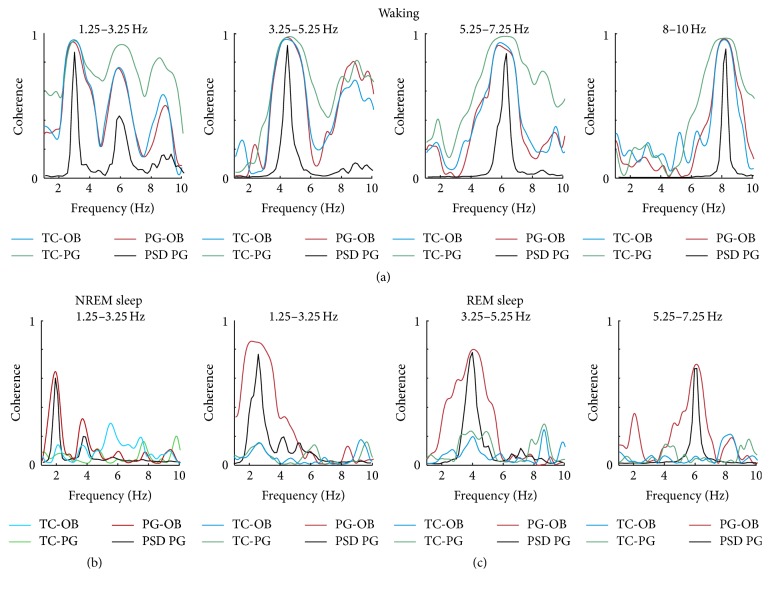
Representative examples of coherences between plethysmography (PG), thermocouple (TC), and olfactory bulb (OB) local field potentials (LFP) reveal clear peaks and their harmonics in waking but only between PG and OB in NREM and REM sleep (see also text for conclusions on OB LFP as source of respiration during sleep). PSD PG: power spectral density of the plethysmography signal.

Figure 6 illustrates representative examples of coherence and its harmonics between all three signals: PG-OB (red), TC-OB (blue), and PG-TC (green) at different ranges of respiration during waking (a), NREM sleep (b), and REM sleep (c). In waking (Figure 6(a)), large peaks of coherence between all signals correspond to respiration frequency (PG PSD: black) indicating the reliability of all three signals to measure respiration. During waking and REM sleep, there is no delta activity in OB; therefore, the LFP is a good indicator of respiration and its peak in PSD corresponds to the respiration rate. However, in NREM sleep, peaks at respiration rate remained only for coherence between PG and OB. Coherence analysis indeed demonstrates that the respiration-related rhythm is preserved in OB during sleep. However, for coherence analysis, a second source of respiration is required during NREM sleep; the OB LFP alone is not sufficient as unequivocal signal of respiration due to multiple equivocal peaks in the power spectral density of the OB LFP.

## 4. Discussion

In the present study, we report slow local field potentials (LFP) in the granular cell layer of olfactory bulb (OB) which reliably indicate respiration in waking mice. This was verified by simultaneous measurement of nasal thermocouple (TC) signals and plethysmography (PG). Our results confirm reports from several decades ago which described slow rhythmic LFPs in the OB related to nasal air flow and breathing [16–18]. At the time, however, these data could not be independently verified due to lack of adequate digital analysis tools. During subsequent years, the concept of respiration-related rhythmic fluctuations of LFPs in OB was progressively replaced by a terminology which subsumed slow OB rhythms as olfaction-related theta oscillations [25–28]; see also discussion in [9]. From a different viewpoint, however, theta oscillations are not just rhythmic fluctuations in the respective frequency range (4–12 Hz) but share the characteristic behavioral features of hippocampal theta rhythms. Theta oscillations occur during locomotion, exploration [29], arousal, or fear [30, 31] or in REM sleep [32]. In contrast, respiration-entrained rhythms in OB, if not masked by delta waves during sleep, are present in any behavioral state. They reflect respiration, and their amplitude is largest during relaxed wakefulness [9, 33], a state when theta rhythm is absent [29, 34]. Indeed, we have recently demonstrated that a clearly distinct respiration-related rhythm (RR) occurs alone or simultaneously with theta oscillations both in OB and in upstream regions like the dentate gyrus of the hippocampal formation [8, 9]. Hippocampal RR is generated by different mechanisms than theta. For example, tracheotomy abolishes RR but not theta oscillations, whereas atropine diminishes theta but does not affect RR [8].

In the present study, we report that only PG, but not TC or OB LFPs, is trustworthy proxy for respiration during NREM sleep. While RR in OB repeatedly returned during NREM and especially during REM sleep, the TC signal was absent throughout NREM and REM sleep. This may be in part explained by a decrease in nasal air flow during these states. Indeed, total inspiratory flow in mice decreases continuously from quiet waking to NREM and REM sleep [35]. Other factors, like redirection of air flow inside the nasal cavity [36], may contribute to the disappearance of respiration signals at the TC. Interestingly, both signals, TC and RR in OB, regularly reappeared during microarousals, providing a potential indicator of these brief states with enhanced vigilance.

In summary, investigating respiration and its related oscillations in the mouse brain during NREM sleep requires PG or other signals different from TC and RR in OB. In waking states of freely moving and behaving mice and in parts of REM sleep with low respiration rates, however, OB LFPs can be reliably used to measure respiration. In addition, we report that RR peak power decreases with increasing respiration rate under several behavioral conditions in wakefulness and REM sleep. Our findings from mice are in good agreement with earlier reports on anesthetized rats [37, 38]. In any case, measuring respiratory rhythm during NREM sleep and separating it from delta activity require great caution.

## 5. Conclusions

Local field potentials in the olfactory bulb of waking but not sleeping mice reliably reflect respiration. This holds also true for nasal thermocouples. In sleeping mice, the most accurate method to record breathing is whole-body plethysmography. Nasal thermocouples are completely unsuitable. Local field potentials from olfactory bulb are reliable in parts of REM sleep with low respiration rates but alone are not sufficient in NREM sleep.
